# Association between the Risk of Preterm Birth and Low Birth Weight with Periodontal Disease in Pregnant Women: An Umbrella Review

**DOI:** 10.3390/dj11030074

**Published:** 2023-03-07

**Authors:** Tania Padilla-Cáceres, Heber Isac Arbildo-Vega, Luz Caballero-Apaza, Fredy Cruzado-Oliva, Vilma Mamani-Cori, Sheyla Cervantes-Alagón, Evelyn Munayco-Pantoja, Saurav Panda, Hernán Vásquez-Rodrigo, Percy Castro-Mejía, Delsi Huaita-Acha

**Affiliations:** 1 Department of General Dentistry, Dentistry School, Universidad Nacional del Altiplano, Puno 21001, Peru; 2 Research Institute in Environmental Sciences, Health and Biodiversity—IICASB, Universidad Nacional del Altiplano, Puno 21001, Peru; 3 Department of General Dentistry, Dentistry School, Universidad San Martín de Porres, Chiclayo 14012, Peru; 4 Department of Human Medicine, School of Human Medicine, Universidad San Martín de Porres, Chiclayo 14012, Peru; 5 Department of General Dentistry, Dentistry School, Universidad Alas Peruanas, Lima 15072, Peru; 6 Department of Nursing, School of Nursing, Universidad Nacional del Altiplano, Puno 21001, Peru; 7 Department of Stomatology, School of Stomatology, Universidad Nacional de Trujillo, Trujillo 13011, Peru; 8 Amazonian Andean Research and Development Institute—IIDEAA, Universidad Nacional del Altiplano, Puno 21001, Peru; 9 Emerge, Emerging Diseases and Climate Change Research Unit, Universidad Peruana Cayetano Heredia, Lima 15102, Peru; 10 Department of Pediatric Stomatology, Dentistry School, Universidad Nacional Mayor de San Marcos, Lima 15081, Peru; 11 Bibliometrics Research Unit, Universidad de San Ignacio de Loyola, Lima 15024, Peru; 12 Department of Periodontics and Oral Implantology, Siksha ‘O’ Anusandhan Univeristy, Bhubaneswar 751003, India; 13 Department of Dentistry, Dentistry School, Universidad Norbert Wiener, Lima 15046, Peru; 14 Department of Engineering and Business, School of Business, Universidad Norbert Wiener, Lima 15046, Peru

**Keywords:** periodontal disease, preterm birth, low birth weight, pregnancy, pregnant women, review

## Abstract

Background: The purpose of this review is to determine the association between the risk of preterm birth and low birth weight in newborns and periodontal disease in pregnant women. Methods: A bibliographic search was carried out until November 2021 in the following biomedical databases: PubMed/Medline, Cochrane Library, Scopus, EMBASE, Web of Science, Scielo, LILACS and Google Scholar. Studies reporting the association between the risk of preterm birth and low birth weight in newborns with periodontal disease in pregnant women, which were systematic reviews, in English and without time limits were included. AMSTAR-2 was used to assess the risk of the included studies, and the GRADEPro GDT tool was used to assess the quality of the evidence and the strength of the recommendation of the results. Results: The preliminary search yielded a total of 161 articles, discarding those that did not meet the selection criteria, leaving only 15 articles. Seven articles were entered into a meta-analysis, and it was found that there is an association between the risk of preterm birth and low birth weight in newborns with periodontal disease in pregnant women. Conclusions: There is an association between the risk of preterm birth and low birth weight in newborns with periodontal disease in pregnant women.

## 1. Introduction

Periodontal disease (PD) is caused by bacteria affecting the supporting structures of the tooth, causing inflammatory processes and the destruction of the periodontium, which can lead to tooth loss [1,2]. This disease is initiated and propagated by an interaction between an altered oral microflora and the host’s vulnerable immune system [3,4]. Gingivitis is the beginning of the disease and is generally due to the accumulation of dental biofilm due to poor oral hygiene. It is characterized by localized inflammation of the gum, which appears red and sometimes bleeding. If gingival disease is not treated, there is a risk that it will progress to chronic periodontitis [5].

This progression of bacterial infection leads to the severe destruction of the periodontium, causing tooth loss, which compromises chewing, aesthetics, self-confidence and quality of life [6]; it also contributes to systemic inflammation, with bacterial substances and inflammatory mediators capable of initiating and promoting systemic diseases [2,7]. The prevalence of PD has been reported to range from 20% to 50% worldwide [8]. There is evidence that PD is associated with heart disease, diabetes mellitus, chronic obstructive pulmonary disease, rheumatoid arthritis and adverse pregnancy outcomes [9].

The prevalence of PD is approximately 40% in pregnant mothers [10]. During pregnancy, due to hormonal factors (high levels of estrogen and progesterone), 50 to 70% of women develop gingivitis, being more vulnerable to PD than their nonpregnant peers [11].

Approximately 15 million premature babies are born each year in the world; these children, in addition, are usually born weighing less than 2500 g [12]. Premature babies or premature birth (PB) with low birth weight (LBW) are one of the leading causes of infant morbidity and mortality. Some risk factors that influence these adverse pregnancy outcomes are multiparity, low socioeconomic status, mother’s age, race, history of PB, maternal infectious processes and alcohol and drug abuse [13,14]. Although more than 60% of PBs occur in Africa and South Asia, preterm birth is a global problem [15].

PBs are a primary public health problem in both developed and developing countries. Despite improvements in obstetric care, preterm birth rates have not decreased in the last ten years [16,17,18].

Some studies have related the presence of PD during pregnancy with PB and/or LBW [19,20,21,22,23,24,25], and the evidence refers to an association of PD with adverse effects in pregnancy. Two pathogenic mechanisms are mentioned to explain the effect of PD on adverse pregnancy outcomes. Firstly, the periodontopathogenic bacteria that are found in the bacterial plaque of the gingiva due to a translocation phenomenon directly affect the fetus by bacteremia [15,26].

Likewise, the inflammatory mediators secreted in the subgingival inflammation zone (IL-1, L-6, IL-8, TNF-alpha, prostaglandin E2) come from the fetoplacental unit and produce an inflammatory reaction [27]. Mothers with PD have a high possibility of giving birth to a baby with LBW, prematurely or both in comparison with a pregnant woman with a healthy periodontium; therefore, they may have seven times the risk of having a PB or LBW baby [22,28]. Therefore, the early detection of PDs in pregnant women will help to prioritize the development of preventive and therapeutic interventions to decrease the occurrence of PBs and LBW newborns [29,30].

Given the relevance of this topic for public health, it is important that, through the analysis of systematic reviews with or without meta-analysis, results are produced that allow for more consistent conclusions to be reached. The main objective of this systematic review is to critically appraise the literature on the association between the risk of PB and LBW in newborns with PD in pregnant women.

## 2. Materials and Methods

### 2.1. Protocol and Registration

This systematic review followed a protocol defined by the authors according to the Preferred Reporting Items for Systematic Reviews and Meta-Analyses (PRISMA) guidelines [31]. This protocol has the registration number CRD42021290027 of the International Prospective Registry of Systematic Reviews (PROSPERO).

To prepare and structure this review, the focused question was formulated using the PICO format (population, intervention, outcomes and results) as detailed below:

- Population: pregnant women with PB and LBW;
- Intervention: pregnant women with PD;
- Comparison: pregnant woman without PD;
- Outcomes: association between PD and PB (<37 weeks) and LBW (<2500 gm) of the newborn.

### 2.2. Focused Question (PICO)

Is there an association between the risk of PB and LBW with PD in pregnant women?

### 2.3. Search and Selection of Studies

For the present systematic review, 8 electronic databases were reviewed (PubMed/Medline, Cochrane Library, Scopus, EMBASE, Web of Science, Scielo, LILACS and Google Scholar) until November 2021, combining keywords and subject titles according to the thesaurus of each database: “periodontal disease”, “periodontitis”, “gingivitis”, “preterm birth”, “low birth weight”, “perinatal outcomes”, “premature labor” and “adverse pregnancy outcomes”. The search strategies of each of the databases are presented in Table 1.

The electronic search was independent in the different databases and was carried out by 4 authors (H.A., T.P., L.C. and F.C.). The final decision for inclusion in the study required that articles be systematic reviews with or without meta-analysis, written in English, without time limits and reporting the association between the risk of PB and LBW with PD in pregnant women. Articles that were prospective studies and unpublished studies were excluded.

### 2.4. Data Extraction

For the extraction of data from the eligible studies, a predefined format was used that included the author(s), year of publication, type of study, type of studies included, number of studies included in the qualitative analysis, number of studies included in quantitative analysis, type of periodontal disease, main results, OR/RR and conclusions. Information was extracted independently by three investigators (V.M., S.C. and E.M.) and any disagreement was resolved by consulting the opinion of a fourth investigator.

### 2.5. Risk of Bias (RoB) Assessment

The RoB of the included studies was independently assessed by two calibrated authors (V.M. and T.P.) (k = 0.98) using AMSTAR-2, which is a critical appraisal tool for systematic reviews of health research studies [32], and all disagreements were resolved by discussion with a fourth reviewer (H.V.). According to this tool, a systematic review is evaluated in 16 domains with simple answer options: “yes” when the result is positive; “no” when the standard was not met or there is insufficient information to answer; and “if partial” in cases where there was partial adherence to the standard. They are then classified into four confidence levels: high, moderate, low and critically low.

### 2.6. Analysis of Results

Extracted data were analyzed in RevMan 5.3 (Cochrane Group, Londres, UK) by OR measurement in a fixed effects model with a 95% confidence interval. Additionally, a GRADE analysis was performed using the guideline development tool (GRADEPro GDT) (McMaster University and Evidence Prime Inc., Canada).

## 3. Results

### 3.1. Selection of Studies

**Figure 1:**
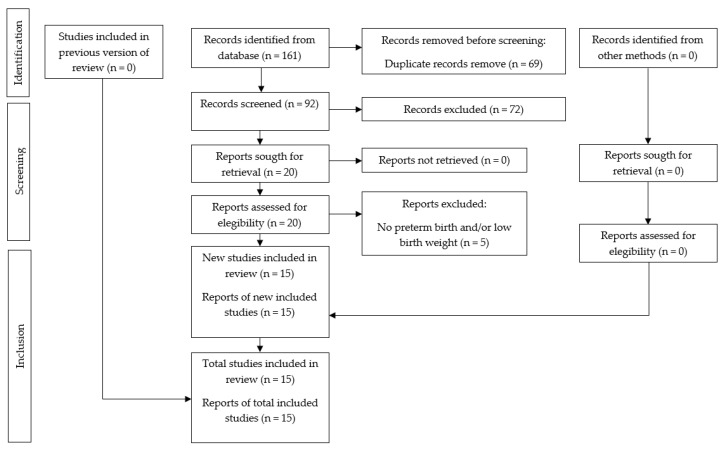
PRISMA flowchart showing the process of inclusion and exclusion of studies in the systematic review.

The strategy used was orientated through electronic and manual search with a total of 161 articles and 69 duplicates (Figure 1). After the selection of titles and abstracts, we chose 20 full-text articles. Then, five studies were excluded, resulting in 15 systematic reviews meeting the eligibility criteria for qualitative synthesis and seven for the quantitative analysis (meta-analysis). The reasons for the exclusion of the studies are found in Table 2.

### 3.2. Characteristics of the Included Studies

Overall, 15 systematic reviews were included [19,20,21,22,23,24,25,38,39,40,41,42,43,44,45], of which nine [19,20,23,38,39,40,41,42,45] had meta-analyses. The following is a list of the countries where the research was conducted: Brazil [25,38], Spain [19], Colombia [20], Indonesia [21], Ethiopia [22], Italy [23,39,45], United Kingdom [40], United States [24,41] and Jordan [42] (Table 3).

All the systematic reviews included studies of various types [19,20,21,22,23,24,25,38,39,40,41,42,43,44,45], such as case–control, cohort, cross-sectional and clinical trials. The number of studies included for qualitative and quantitative analysis ranged from five to thirty-six and from four to twenty-two, respectively. All the studies [19,20,21,22,23,24,25,38,39,40,41,42,43,44,45] evaluated PD, and four studies [21,22,38,44] had LBW as the main outcome, one study [20] PB, six studies [19,24,25,41,43,45] PB and LBW, one study [40] PB and PB with LBW and three studies [23,39,42] PB, LBW and PB with LBW (Table 3).

The composition of the studies with meta-analyses was [19,20,23,38,39,40,41,42,45] two studies [41,45] performed the meta-analysis based solely on clinical trials, five studies [20,23,38,40,42] took the odds ratio as a measure of association and three studies [19,39,40] took the relative risk as a measure of association. Only three studies [25,44,45] concluded that it was not possible to determine the association between PD with PB and LBW (Table 3).

### 3.3. Risk of Bias in the Analysis of the Studies

Eight studies [19,20,22,23,38,39,40,41] had a high overall confidence, five [21,24,25,43,44] had a moderate overall confidence and two [42,45] had a low overall confidence (Table 4).

### 3.4. Synthesis of the Results

**Figure 2:**
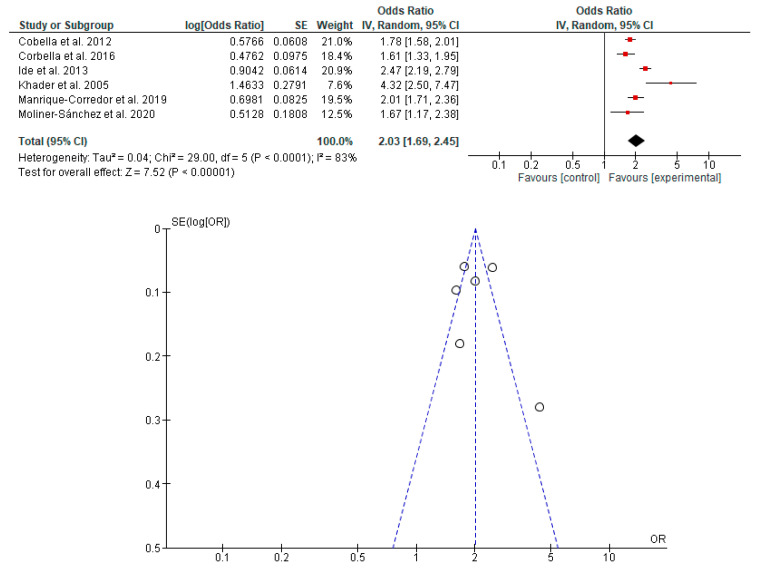
Forest plot and funnel plot of the association between the risk of PB in newborns with PD in pregnant women.

**Figure 3:**
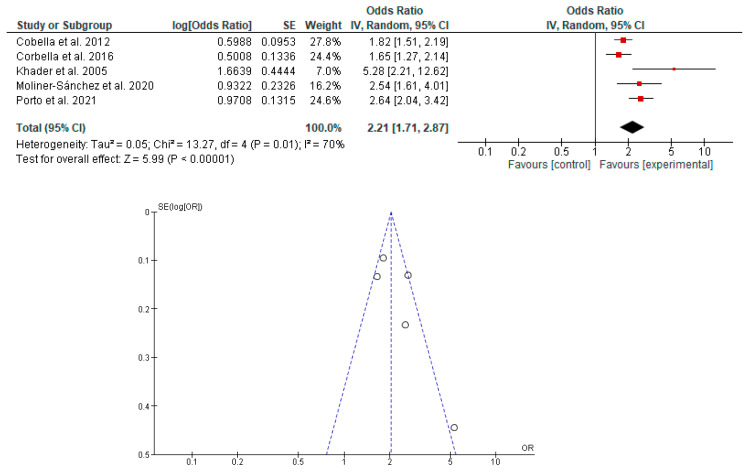
Forest plot and funnel plot of the association between the risk of LBW in newborns with PD in pregnant women.

**Figure 4:**
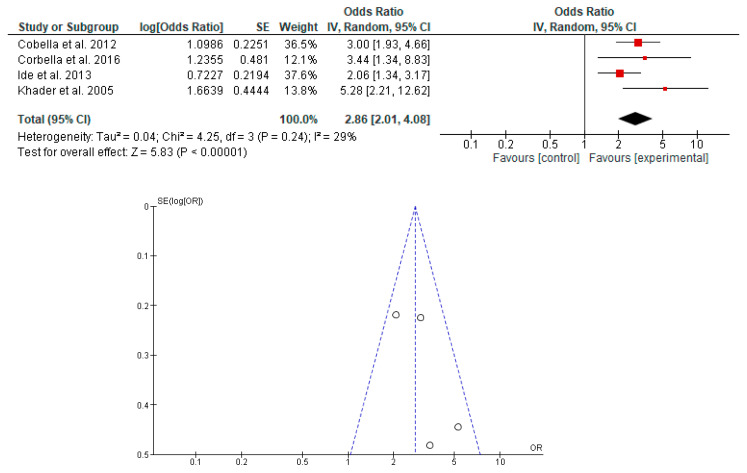
Forest plot and funnel plot of the association between the risk of PB with LBW in newborns with PD in pregnant women.

The association between the risk of PB and LBW in newborns with PD in pregnant women was determined in seven studies [19,20,23,38,39,40,42]. It was shown that PD in pregnant women is associated with the risk of PB, LBW and PB with LBW in newborns (Figure 2, Figure 3 and Figure 4).

### 3.5. GRADE Analysis

When evaluating the included studies, it was observed that there is low and moderate certainty in the association of PD in pregnant women with PB and LBW in newborns (Table 5).

## 4. Discussion

Diagnoses, treatments and clinical decisions in dentistry must be based on the best available scientific evidence, which comes from systematic reviews.

One of the objectives is to analyze whether these systematic reviews had an optimal design and execution process. For this, the AMSTAR tool was developed, which was introduced for the first time in 2007 [32,46,47]. To increase the applicability of the AMSTAR tool, a group of experts made reviews and created the AMSTAR-2 tool [32,47], which was used in the present review.

The main problem found was that one of the studies [42] did not use an exhaustive literature search strategy, and another study [45] did not present the list of excluded studies. Several protocols, such as those found in Cochrane systematic reviews, recommend that authors provide lists of included and excluded articles, allowing the reader to easily judge the quality of the selected articles.

Additionally, some studies [21,24,25,43,44] did not meet certain points for high overall confidence; for example, they did not perform duplicate study selection and data extraction. These are important issues that authors of systematic reviews should pay attention to in the future.

In the present study, it was observed that there is an association between the risk of PB and LBW in newborns with PD in pregnant women. This may be due to inflammatory processes occurring at the placenta–fetus junction or elevated systemic inflammation in pregnant women or due to the translocation of periodontopathogenic bacteria to the uteroplacental circulation [48].

The World Health Organization (WHO) established, as one of its goals, the reduction in the incidence of PB and LBW of newborns given the great impact that these problems have on children’s morbidity and mortality indicators. In this sense, the efforts that health teams must make to reduce the incidence of PB and LBW of newborns not only aim to reduce maternal–fetal consequences but also the costs of hospitalization, use of units of intensive care, care and prevention in long-term health [49,50,51], as well as the implementation of preventive care protocols during the gestation period to reduce bacterial plaque rates from first care to subsequent visits [52]. Therefore, the authors emphasize the association found in this review so that the necessary public health measures can be taken.

However, this review has some limitations, such as the use of the AMSTAR-2 tool, which only considers the general confidence of the systematic reviews and their elements but does not describe this confidence in the studies included in each systematic review. In addition, the studies included in the systematic reviews that were able to determine the association between PD in pregnant women with PB and LBW had different study designs.

However, it has several important strengths, such as the fact that the present review cannot be compared with other previous studies since an umbrella review has not been previously carried out using the AMSTAR-2 tool. An exhaustive search of the scientific literature was carried out in the main bibliometric search engines, and a result was obtained based on systematic reviews with meta-analyses with high general confidence.

## 5. Conclusions

The systematic reviews included in the present study showed, in general, high confidence. In addition, there is an association between the risk of PB and LBW in newborns born and mothers with PD. A pregnant woman with PD is two to three times more likely to have a PB and a LBW.

## Figures and Tables

**Table 1:** Search strategies for each database.

Database	Search Strategy
PubMed/Medline	(((“periodontal disease treatment”) OR periodontitis) OR gingivitis) AND (((((“preterm birth”) OR “low birth weight”) OR “perinatal outcomes”) OR “premature labor”) OR “adverse pregnancy outcomes”)
Cochrane Library	#1 MeSH descriptor: [Periodontal Diseases] explode all trees #2 MeSH descriptor: [Periodontitis] explode all trees #3 MeSH descriptor: [Gingivitis] explode all trees #4 (periodontal disease treatment) OR (periodontitis) OR (gingivitis) (Word variations have been searched) #5 #1 OR #2 OR #3 OR #4 #6 MeSH descriptor: [Premature Birth] explode all trees #7 MeSH descriptor: [Infant, Low Birth Weight] explode all trees #8 MeSH descriptor: [Obstetric Labor, Premature] explode all trees #9 (preterm birth) OR (low birth weight) OR (perinatal outcomes) OR (premature labor) OR (adverse pregnancy outcomes) (Word variations have been searched) #10 #6 OR #7 OR #8 OR #9 #11 #5 AND #10
Scopus	TITLE-ABS-KEY (((“periodontal disease treatment”) OR periodontitis) OR gingivitis) AND TITLE-ABS-KEY (((((“preterm birth”) OR “low birth weight”) OR “perinatal outcomes”) OR “premature labor”) OR “adverse pregnancy outcomes”) AND TITLE-ABS-KEY (“systematic review”) AND (LIMIT-TO (SUBJAREA, “DENT”)) AND (LIMIT-TO (DOCTYPE, “re”))
Scielo	((((“periodontal disease”) OR (treatment) (periodontitis) OR (gingivitis))) AND (((“preterm birth”) OR (“low birth weight”) OR (“perinatal outcomes”) OR (“premature labor”) OR (“adverse pregnancy outcomes”)))) AND ((“systematic review”)) ((((“enfermedad periodontal”) OR (tratamiento) OR (periodontitis) OR (gingivitis))) AND (((“parto pretérmino”) OR (“bajo peso al nacer”) OR (“resultados perinatales”) OR (“resultados adversos al embarazo”)))) AND ((“revisión sistemática”))
EMBASE	(‘periodontal disease treatment’:ti,ab,kw OR periodontitis:ti,ab,kw OR gingivitis:ti,ab,kw) AND (‘preterm birth’:ti,ab,kw OR ‘low birth weight’:ti,ab,kw OR ‘perinatal outcomes’:ti,ab,kw OR ‘premature labor’:ti,ab,kw OR ‘adverse pregnancy outcomes’:ti,ab,kw) AND ‘systematic review’:ti,ab,kw
Web of Science	((TS = (((“periodontal disease treatment”) OR (periodontitis) OR (gingivitis)))) AND TS = (((“preterm birth”) OR (“low birth weight”) OR (“perinatal outcomes”) OR (“premature labor”) OR (“adverse pregnancy outcomes”)))) AND TS = ((“systematic review”))
Google Scholar	allintitle: “periodontal disease treatment” “preterm birth” “systematic review” allintitle: “periodontitis” “preterm birth” “systematic review” allintitle: “periodontitis” “low birth weight” “systematic review”

**Table 2:** Reason for exclusion of included studies.

Authors	Reason for Exclusion
Bashir et al. [33], Gharehghani et al. [34], Konopka et al. [35], Abariga et al. [36], Kunnen et al. [37]	Studies not referring to PB or LBW

**Table 3:** Characteristics of the included studies.

Author	Year	Type of Study	Country	Type of Studies Included	Number of Studies Included in Qualitative Analysis	Number of Studies Included in Quantitative Analysis	Type of Periodontal Disease	Outcomes	OR/RR	Conclusions
Porto et al. [38]	2021	SR with MA	Brazil	Cases and controls and cohorts	21	21	Periodontitis	LBW	2.64 (2.04–3.42)/NR	Pregnant women with periodontitis may be more than twice as likely to have babies with PB.
Moliner-Sánchez et al. [19]	2020	SR with MA	Spain	Cohorts	11	11 (11 PB and 6 LBW)	Periodontal disease	PB and LBW	NR/PB = 1.67 (1.17–2.38), LBW = 2.54 (1.61–3.98)	There is a relationship between PD and PB and LBW.
Manrique-Corredor et al. [20]	2019	SR with MA	Colombia	Cases and controls and cohorts	31	20	Periodontitis	PB	2.01 (1.71–2.36)/NR	There is an association between periodontitis and PB.
Syafar et al. [21]	2019	SR	Indonesia	Cases and controls	6	0	Periodontal disease	LBW	NR/NR	There is an association between periodontitis and PB.
Teshome et al. [22]	2016	SR	Ethiopia	Cases and controls	10	0	Periodontal disease	LBW	NR/NR	There is an association between periodontitis and PB.
Corbella et al. [39]	2016	SR with MA	Italy	Cases and controls	22	22 (14 PB and 10 LBW)	Periodontitis	PB, LBW and PB + LBW	NR/PB = 1.61 (1.33–1.95), LBW = 1.65 (1.27–2.14), PB + LBW = 3.44 (1.34–8.8)	There is an association between periodontitis and PB, LBW and PB + LBW.
Ide et al. [40]	2013	SR with MA	United Kingdom	Cases and controls, cohorts and cross-sectional	18	16 (14 PB and 2 LBW)	Periodontitis	PB and PB + LBW	PB = 2.47 (2.19–2.77), PB + LBW = 2.06 (1.34–3.16)/PB = 1.15 (0.89–1.49)	There is an association between periodontitis and PB and PB + LBW.
Corbella et al. [23]	2012	SR with MA	Italy	Cases and controls	17	17 (14 PB and 7 LBW)	Periodontal disease	PB, LBW and PB + LBW	PB = 1.78 (1.58–2.01), LBW = 1.82 (1.51–2.2), PB + LBW = 3.00 (1.93–4.68)/NR	There is an association between PD and PB, LBW and PB + LBW.
Corbella et al. [45]	2012	SR with MA	Italy	Cases and controls, cohorts and clinical trials	37	5 (5 PB and 5 LBW)	Periodontal disease	PB and LBW	NR/NR	There was no clear evidence that PD is a major risk factor for adverse pregnancy outcomes, although it may have a minor effect.
Xiong et al. [41]	2007	SR with MA	United States	Cases and controls, cohorts and clinical trials	26 (12 PB and 10 LBW)	4 (3 PB and 2 LBW)	Periodontal disease	PB and LBW	NR/NR	There is an association between PD and PB and LBW.
Vettore et al. [25]	2006	SR	Brazil	Cases and controls, cohorts and clinical trials	36 (30 PB and 28 LBW)	0	Periodontal disease	PB and LBW	NR/NR	It is not possible to determine the association between PD and PB and LBW.
Xiong et al. [24]	2006	SR	United States	Cases and controls, cohorts and clinical trials	25 (8 PB and 6 LBW)	0	Periodontal disease	PB and LBW	NR/NR	There is an association between PD and PB and LBW.
Khader et al. [42]	2005	SR with MA	Jordan	Cases and controls and cohorts	5	4 (3 PB and 2 LBW)	Periodontal disease	PB, LBW and PB + LBW	PB = 4.32 (2.5–7.44), LBW = 5.28 (2.21–12.62), PB + LBW = 5.28 (2.21–12.62)/NR	There is an association between PD and PB, LBW and PB + LBW.
Scannapieco et al. [43]	2003	SR	United States	Cases and controls, cohorts, cross-sectional and clinical trials	12	0	Periodontal disease	PB and LBW	NR/NR	There is an association between PD and PB and LBW.
Madianos et al. [44]	2002	SR	United States	Cases and controls, cohorts, cross-sectional and clinical trials	25 (5 LBW)	0	Periodontitis	PB	NR/NR	The association between PD and PB cannot be determined.

NR: not reported, SR: systematic review, MA: meta-analysis, PD: periodontal disease, PB: preterm birth, LBW: low birth weight.

**Table 4:** Risk of bias in the analysis of the included studies.

Author	Year	AMSTAR-2																Overall Confidence
		1	2 *	3	4 *	5	6	7 *	8	9 *	10	11 *	12	13 *	14	15 *	16	
Porto et al. [38]	2021	Yes	Yes partial	Yes	Yes	Yes	Yes	Yes partial	Yes	Yes	Yes	Yes	Yes	Yes	Yes	Yes	Yes	High
Moliner-Sánchez et al. [19]	2020	Yes	Yes	Yes	Yes	Yes	No	Yes partial	Yes	Yes	Yes	Yes	Yes	Yes	Yes	Yes	Yes	High
Manrique-Corredor et al. [20]	2019	Yes	Yes	Yes	Yes	Yes	Yes	Yes partial	Yes	Yes	No	Yes	Yes	Yes	Yes	Yes	Yes	High
Syafar et al. [21]	2019	Yes	Yes	No	Yes	Yes	Yes	Yes partial	Yes	Yes	No	No meta-analysis	No meta-analysis	Yes	Yes	No meta-analysis	Yes	Moderate
Teshome et al. [22]	2016	Yes	Yes	Yes	Yes	Yes	Yes	Yes partial	Yes	Yes	No	No meta-analysis	No meta-analysis	Yes	Yes	No meta-analysis	Yes	High
Corbella et al. [39]	2016	Yes	Yes	Yes	Yes	Yes	Yes	Yes partial	Yes	Yes	No	Yes	Yes	Yes	Yes	Yes	Yes	High
Ide et al. [40]	2013	Yes	Yes	Yes	Yes	Yes	Yes	Yes partial	Yes	Yes	Yes	Yes	Yes	Yes	Yes	Yes	Yes	High
Corbella et al. [23]	2012	Yes	Yes	Yes	Yes	Yes	Yes	Yes	Yes	Yes	Yes	Yes	Yes	Yes	Yes	Yes	Yes	High
Corbella et al. [45]	2012	Yes	Yes partial	Yes	Yes	Yes	No	No	Yes	Yes	No	Yes	Yes	Yes	Yes	Yes	Yes	Low
Xiong et al. [41]	2007	Yes	Yes	Yes	Yes	Yes	Yes	Yes partial	Yes	Yes	Yes	Yes	Yes	Yes	Yes	Yes	Yes	High
Vettore et al. [25]	2006	Yes	Yes partial	Yes	Yes	No	No	Yes partial	Yes	Yes	Yes	No meta-analysis	No meta-analysis	Yes	Yes	No meta-analysis	Yes	Moderate
Xiong et al. [24]	2006	Yes	Yes	Yes	Yes	No	No	Yes partial	Yes	Yes	No	No meta-analysis	No meta-analysis	Yes	Yes	No meta-analysis	No	Moderate
Khader et al. [42]	2005	Yes	Yes partial	Yes	No	Yes	Yes	Yes partial	Yes	Yes	Yes	Yes	Yes	Yes	Yes	Yes	No	Low
Scannapieco et al. [43]	2003	Yes	Yes	Yes	Yes	Yes	Yes	Yes partial	Yes	Yes	No	No meta-analysis	No meta-analysis	Yes	Yes	No meta-analysis	No	Moderate
Madianos et al. [44]	2002	Yes	Yes partial	Yes	Yes	Yes	Yes	Yes partial	Yes	Yes	No	No meta-analysis	No meta-analysis	Yes	Yes	No meta-analysis	No	Moderate

AMSTAR = Measurement tool that evaluates systematic reviews. AMSTAR-2 items: (i) Do the research questions and inclusion criteria for the review include PICO components? (ii) Does the review report include an explicit statement that the review methods were established prior to the execution and justify any significant deviations from the protocol? (iii) Did the reviewers state their decision on the study designs to include in the review? (iv) Did the reviewers apply a comprehensive literature search strategy? (v) Did the reviewers run the study selection in duplicate? (vi) Did the study authors perform data extraction in duplicate? (vii) Did the reviewers provide and justify a list of excluded studies? (viii) Did the reviewers describe the included studies in sufficient detail? (ix) Did the review authors use a satisfactory technique to assess the risk of bias in the individual studies included in the review? (x) Did the review authors report the sources of funding for the studies included in the review? (xi) If a meta-analysis was performed, did the review authors use appropriate methods for the statistical combination of results? (xii) If a meta-analysis was performed, did the review authors assess the potential impact of risk of bias in individual studies on the results of the meta-analysis or other evidence synthesis? (xiii) Did the review authors consider the risk of bias of individual studies when interpreting/discussing the results of the review? (xiv) Did the review authors provide a satisfactory explanation and discuss any observed heterogeneity in the review results? (xv) Quantitative synthesis completed, did reviewers adequately investigate publication bias (small study bias) and discuss its likely impact on review results? (xvi) Did the review authors report any potential sources of conflict of interest, including any funding received to carry out the review? * = critical domain.

**Table 5:** GRADE analysis.

Certainty Assessment							Certainty
№ of Studies	Study Design	Risk of Bias	Inconsistency	Indirectness	Imprecision	Other Considerations	
Association between the risk of PB in newborns with PD in pregnant women							
6	SR	not serious	very serious	not serious	not serious	none	⨁⨁◯◯ Low
Association between the risk of LBW in newborns with PD in pregnant women							
5	SR	not serious	very serious	not serious	not serious	none	⨁⨁◯◯ Low
Association between the risk of PB with LBW in newborns with PD in pregnant women							
4	SR	not serious	serious	not serious	not serious	none	⨁⨁⨁◯ Moderate

SR: Systematic review.

## Data Availability

Not applicable.
